# Supplementary material for: Sulfonated PAM/PPy Cryogels with Lowered Evaporation Enthalpy for Highly Efficient Photothermal Water Evaporation
Source: Polymers (Basel). 2023 Apr 28;15(9):2108. doi: 10.3390/polym15092108 (PMC10180751; doi:10.3390/polym15092108)
Supplement: Supplementary file 1 [file polymers-15-02108-s001.zip › polymers-2289693-supplementary.pdf]

polymers

MDPI

Supplementary Materials

Sulfonated PPy/PAAm Cryogels with Low Evaporation Enthalpy for Highly Efficient Photothermal Water Evaporation

Shi-Chang Hou [1], Dao-Wei Zhang [1], Jun Chen [1], Xiao-Xiao Guo [1], Abdul Haleem [2] and Wei-Dong He [1,*]

[1] CAS Key Laboratory of Soft Matter Chemistry, Department of Polymer Science and Engineering, University of Science and Technology of China, Hefei 230026, China; housc@mail.ustc.edu.cn (S.-C.H.); cdw88@mail.ustc.edu.cn (D.-W.Z.); junchen9@mail.ustc.edu.cn (J.C.); sa05@mail.ustc.edu.cn (X.-X.G.)

[2] School of Chemistry and Chemical Engineering, Jiangsu University, Zhenjiang 212013, China; haleem@mail.ustc.edu.cn

* Correspondence: wdhe@ustc.edu.cn

Citation: Hou, S.-C.; Zhang, D.-W.; Chen, J.; Guo, X.-X.; Haleem, A.; He, W.-D. Sulfonated PAM/PPy Cryogels with Lowered Evaporation Enthalpy for Highly Efficient Photothermal Water Evaporation. *Polymers* **2023**, *15*, 2108. https://doi.org/10.3390/polym15092108

Academic Editor: Łukasz Klapiszewski

Received: 3 March 2023
Revised: 25 April 2023
Accepted: 26 April 2023
Published: 28 April 2023

Copyright: © 2023 by the authors. Licensee MDPI, Basel, Switzerland. This article is an open access article distributed under the terms and conditions of the Creative Commons Attribution (CC BY) license (https://creativecommons.org/licenses/by/4.0/).

Determination of equivalent evaporation enthalpy of water in PAM cryogels

One two-layer iron container was used for the measurement as shown Figure S1. The test samples of pure water or different swollen cryogels were placed in a petri dish shelved on the upper plate and anhydrous calcium chloride in a glass beaker was placed at the button. After that, the iron container was capped, sealed with paraffin film and kept in a thermostatic bath. The temperature in the thermostatic bath and inside the container was measured with a thermal couple. After certain period, the samples were taken out and weight.

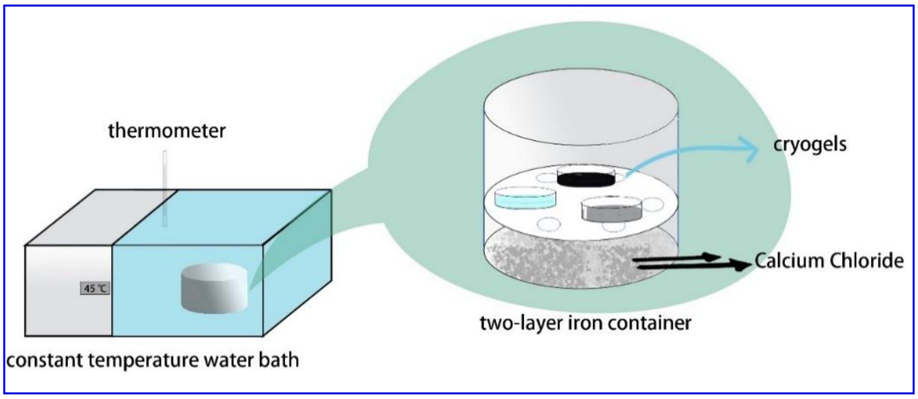

Figure S1. Measurement system for equivalent enthalpy of evaporation of water in PAM cryogels.

Composition comparison of sulfonated PAM before and after PPy loading

As shown in Figure S2 was FTIR spectra of PAM-S(0.2) cryogels before and after loading polypyrrole. It is evident that PAM-S(0.2)@PPy has FTIR signals at 1194 cm^{-1} and 928 cm^{-1} ascribed to =C-H plane vibration and C-H in-plane deformation vibration of PPy [1, 2], respectively.

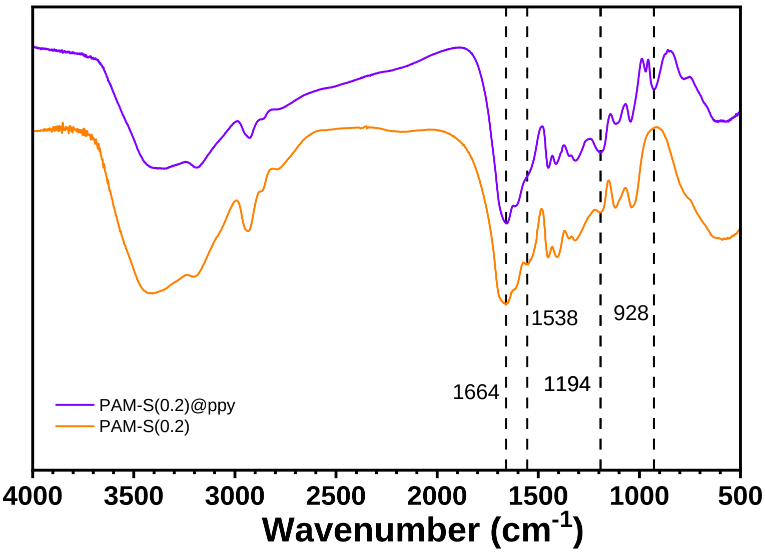

Figure S2. FTIR spectra of PAM-S(0.2) and PAM-S(0.2)@PPy cryogels.

Molar percent of sulfonated AM unit in the crosslinked polyacrylamide

The molar percent of sulfonated acrylamide (AM) unit in the crosslinked PAM was coarsely calculated accroding to the following equation, taking one MBAm residual unit as two AAm residual units. Additonally, the molar percent of sulfur element was also calculated based on the mass percents of all elements (C, H, N and O). The resutls are listed in Table S1.

$$\mathrm{mol_s} = \frac{\omega_1/\mathrm{Ar_1}}{(1-\omega_1/\mathrm{Ar_1}\cdot\mathrm{Mr_1})/\mathrm{Mr_2}} \times 100\% \quad \text{(S1)}$$

where ω_1 is the mass percent of sulfur, and Ar_1 is the relative atomic mass of sulfur atom, Mr_1 and Mr_2 are the relative molecular mass of sulfonic acid groups and acrylamide unit, respectively.

Table S1. The content of sulfur element in PAM and sulfonated PAM cryogels.

Sample	S (wt %)	S (mol %)	sulfonated AM unit (mol %)[a]
PAM-S(0)	0.012	0.001	0.026
PAM-S(0.1)	1.960	0.061	4.247
PAM-S(0.2)	3.657	0.114	7.767
PAM-S(0.3)	5.876	0.184	12.166
PAM-S(0.5)	7.644	0.239	15.515
PAM-S(0.2)@PPy	2.965	0.093	6.349 in PAM and PPy

Morphology comparison of sulfonated PAM before and after PPy loading

By the comparison of SEM image of PAM-S(0)@PPy (Figure S3a)with others (Figure S3b-d), the cryogel surface become rougher with more wrinkles, suggesting PPy might covered more cryogel surface with the increase of sulfonation degree.

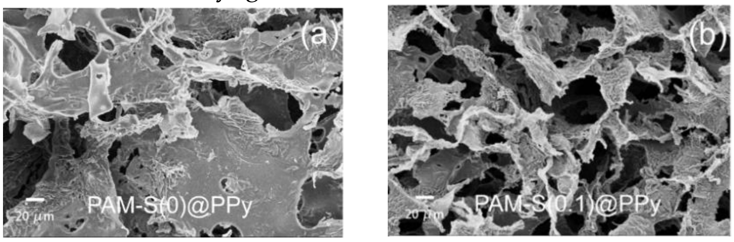

(**a**) (**b**)

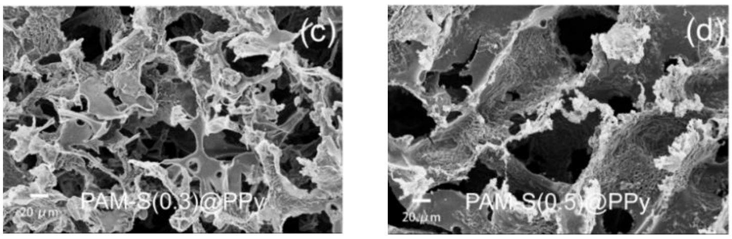

(c) (d)

Figure S3. SEM images of PAM-S(0)@PPy (**a**), PAM-S(0.1)@PPy (**b**), PAM-S(0.3)@PPy (**c**) and PAM-S(0.5)@PPy cryogels (**d**).

Morphology comparison of sulfonated PAM before and after PPy loading

The contact angle of cryogel against water was measured by a contact angle measuring device with the aid of a high-speed camera, since the high porosity and hydrophilicity of those cryogels caused the immediate penetration of water drop into the cryogel interior. Table S2 listed the dynamic contact angle at 0.01 sec after dripping water drop onto cryogel surface.

Table S2. Dynamic contact angle at 0.01 s and solar light absorption efficiency (η_{abs}).

Sample	contact angle (°)	η_{abs} (%)
PAM-S(0)@PPy	29.0	97.94
PAM-S(0.1)@PPy	83.3	98.29
PAM-S(0.2)@PPy	101.3	98.42
PAM-S(0.3)@PPy	109.4	97.56
PAM-S(0.5)@PPy	128.5	98.35

TGA results of sulfonated PAM before and after PPy loading

As shown in Figure S4, as the sulfonation degree increased, the first decomposition temperature of PAM-S cryogels decreased from 220.2 °C [a: PAM-S(0)] to 145.8 °C [e: PAM-S(0.5)] due to the presence of imide group with the bonded methylenesulfonate. After PAM-S(0.2) (c) was loaded with polypyrrole, the first decomposition temperature decreased from 159.3 °C to 141.1 °C [f: PAM-S(0.5)@PPy], caused by the loss of HCl dopant.

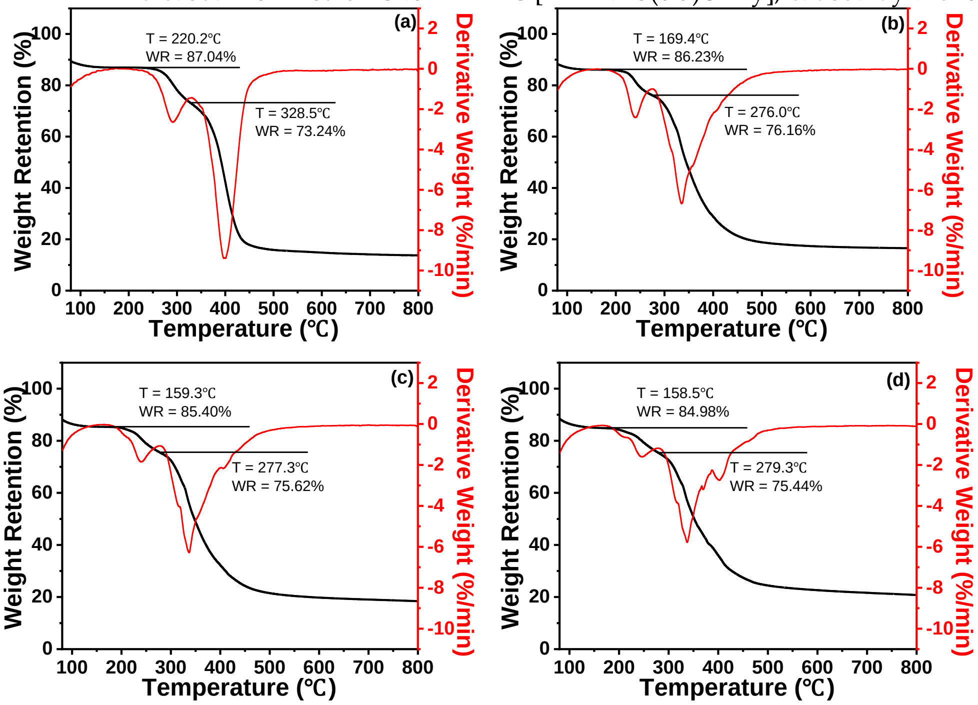

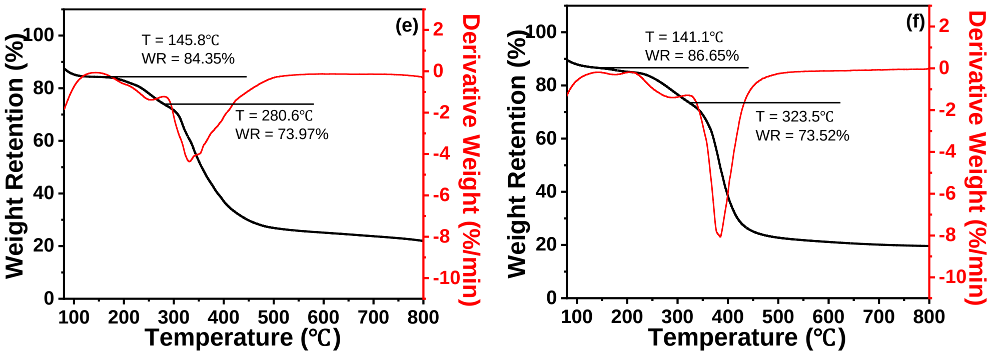

Figure S4. Thermogravimetric analysis results of PAM-S(0) (a), PAM-S(0.2) (b), PAM-S(0.2) (c), PAM-S(0.3) (d), PAM-S(0.5) (e) and PAM-S(0.2)@PPy (f).

Temperature recording of cryogel surface before photothermal evaporation

Before photothermal evaporation, the surface temperature of different cryogels was recorded with infrared camera to have the initial temperature. The infrared camera photos are shown in Figure S5. As well, their values as well as the temperature during photothermal evaporation are summarized in Table S1.

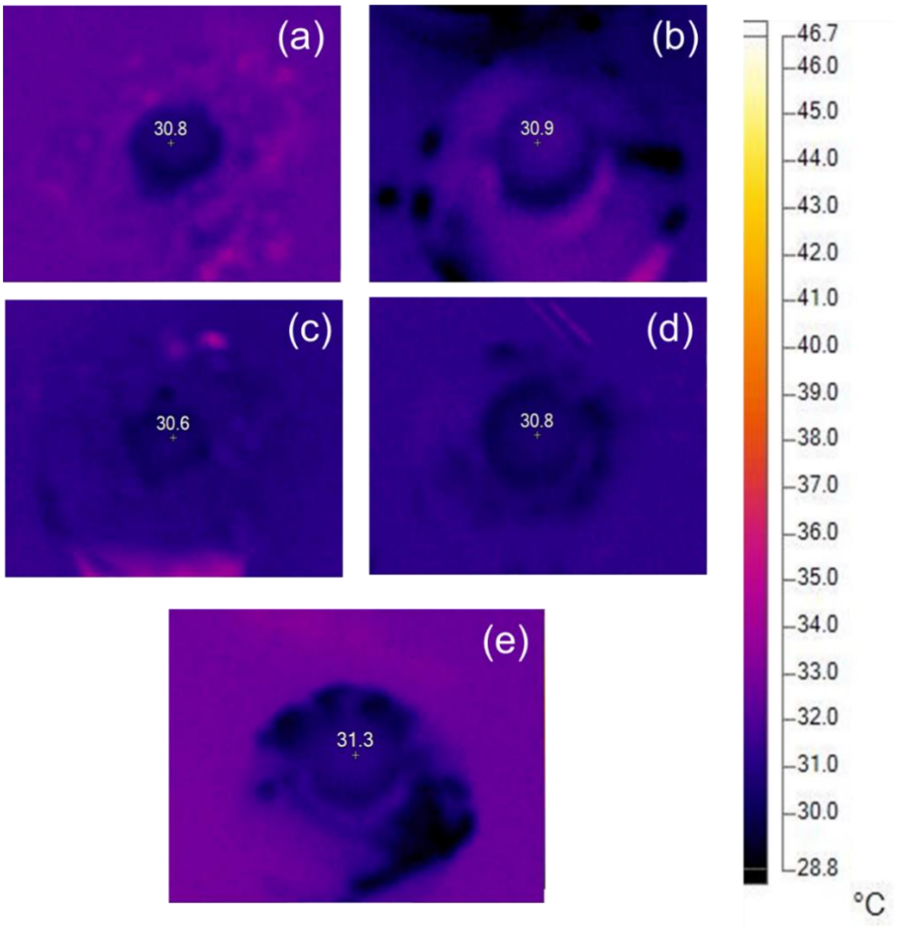

Figure S5. Infrared camera photos of PAM-S(0)@PPy (a), PAM-S(0.1)@PPy (b), PAM-S(0.2)@PPy (c), PAM-S(0.3)@PPy (d) and PAM-S(0.5)@PPy (e) cryogel surface before the photothermal evaporation.

Table S3. The solar light absorption efficiency, the temperature change of different cryogels under one-sun irradiation during photothermal evaporation and their contact angles.

Sample	η_{abs} (%)	$T_{initial}$ (℃)	T_{stable} (℃)	ΔT(℃)	contact angle (°)

PAM-S(0)@PPy	97.94	30.8	42.7	11.9	29.0
PAM-S(0.1)@PPy	98.29	30.9	41.7	10.8	83.3
PAM-S(0.2)@PPy	98.42	30.6	42.6	12.0	101.3
PAM-S(0.3)@PPy	97.56	30.8	44.2	13.4	109.4
PAM-S(0.5)@PPy	98.35	31.3	44.1	12.8	128.5

Salting-out observation during photothermal evaporation

Photothermal evaporation of 14 wt% NaCl solution was carried out with PAM-S(0.2)@PPy under one-sun irradiation. In each day, the evaporation lasted for 5 h and the surface was observed with a digital camera, as shown in Figure S6. It is seen that no salt appeared at the cryogel surface at the end of the third day.

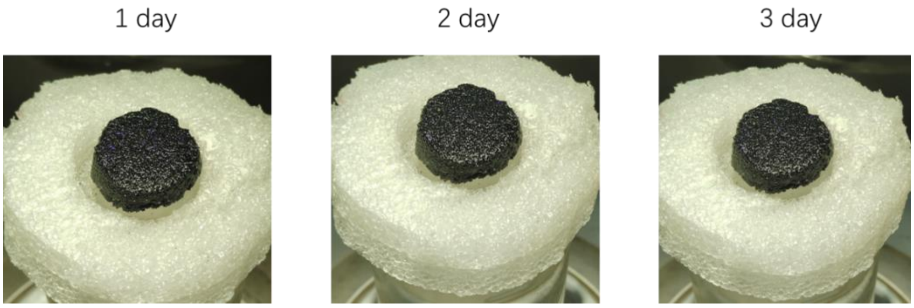

Figure S6. No apparent salt crystallization on the surface of the cryogel during three days of repeated evaporation of high-concentration saline for 5 h.

References

1) Omastová, M.; Trchová, M.; Kovářová, J.; Stejskal, J. Synthesis and structural study of polypyrroles prepared in the presence of surfactants. *Synth. Met.* **2003**, *138*, 447–455, https://doi.org/10.1016/s0379-6779(02)00498-8.
2) Blinova, N.V.; Stejskal, J.; Trchová, M.; Prokeš, J.; Omastová, M. Polyaniline and polypyrrole: A comparative study of the preparation. *Eur. Polym. J.* **2007**, *43*, 2331–2341, https://doi.org/10.1016/j.eurpolymj.2007.03.045.

Disclaimer/Publisher's Note: The statements, opinions and data contained in all publications are solely those of the individual author(s) and contributor(s) and not of MDPI and/or the editor(s). MDPI and/or the editor(s) disclaim responsibility for any injury to people or property resulting from any ideas, methods, instructions or products referred to in the content.
